# Documentation of adherence to infection prevention best practice in patient records: a mixed-methods investigation

**DOI:** 10.1186/s13756-022-01139-2

**Published:** 2022-08-25

**Authors:** Alen Hascic, Aline Wolfensberger, Lauren Clack, Peter W. Schreiber, Stefan P. Kuster, Hugo Sax

**Affiliations:** 1 Department of Infectious Diseases and Hospital Epidemiology, University Hospital Zurich, University of Zurich, Zurich, Switzerland; 2 Institute for Implementation Science in Health Care, Medical Faculty, University of Zurich, Zurich, Switzerland; 3 Present Address: Division of Infectious Diseases and Hospital Epidemiology, Cantonal Hospital St. Gallen, St. Gallen, Switzerland; 4 Present Address: Department of Infectious Diseases, Bern University Hospital and University of Bern, Friedbuehlstrasse 53, 3010 Bern, Switzerland

**Keywords:** Infection prevention, Infection control, Healthcare-associated infections, Prevalence study, Mixed-method research, Documentation

## Abstract

**Background:**

Healthcare-associated infections remain a preventable cause of patient harm in healthcare. Full documentation of adherence to evidence-based best practices for each patient can support monitoring and promotion of infection prevention measures. Thus, we reviewed the extent, nature, and determinants of the documentation of infection prevention (IP) standards in patients with HAI.

**Methods:**

We reviewed electronic patient records (EMRs) of patients included in four annual point-prevalence studies 2013–2016 who developed a device- or procedure-related HAI (surgical site infection (SSI), catheter-associated urinary tract infection (CAUTI), ventilator-associated infection (VAP), catheter-related bloodstream infection (CRBSI)). We examined the documentation quality of mandatory preventive measures published as institutional IP standards. Additionally, we undertook semi-structured interviews with healthcare providers and a two-step inductive (grounded theory) and deductive (Theory of Planned Behaviour) content analysis.

**Results:**

Of overall 2972 surveyed patients, 249 (8.4%) patients developed 272 healthcare-associated infections. Of these, 116 patients met the inclusion criteria, classified as patients with SSI, CAUTI, VAP, CRBSI in 78 (67%), 21 (18%), 10 (9%), 7 (6%), cases, respectively. We found documentation of IP measures in EMRs in 432/1308 (33%) cases. Documentation of execution existed in the study patients’ EMRs for SSI, CAUTI, VAP, CRBSI, and overall, in 261/931 (28%), 27/104 (26%), 46/122 (38%), 26/151 (17%), and 360/1308 (28%) cases, respectively, and documentation of non-execution in 67/931 (7%), 2/104 (2%), 0/122 (0%), 3/151 (2%), and 72/1308 (6%) cases, respectively. Healthcare provider attitudes, subjective norms, and perceived behavioural control indicated reluctance to document IP standards.

**Conclusions:**

EMRs rarely included conclusive data about adherence to IP standards. Documentation had to be established indirectly through data captured for other reasons. Mandatory institutional documentation protocols or technically automated documentation may be necessary to address such shortcomings in patient safety documentation.

## Background

The prevention of healthcare-associated infections (HAI) represents a conundrum in healthcare delivery worldwide and after decades of infection prevention and control efforts in most hospitals, the preventable proportion of HAI still ranges between 35 and 55% [1–3].

This study began with the idea of identifying the potential for preventing HAI in individual patients. It has been argued—for promotional purposes—that HAI in individual patients for which the evidence-based prevention practices have not been applied, should be declared as preventable [4]. To benefit from this promotional effect—and to investigate the causality for HAI in individual patients and care settings –, it would be necessary to dispose of a seamless documentation of the execution or non-execution of established prevention measures in their electronic medical record (EMR). Moreover, given the potentially severe consequences of HAI, patients might have an invested interest to know if all prevention measures were applied during their hospital stay.

Thus, we conducted a mixed-methods study. First, to investigate completeness of the documentation quality of infection prevention (IP) measures, we quantitatively scrutinized the EMRs of a defined patient population who suffered from one of the four major procedure-related HAI. Second, to better understand barriers and opportunities for improvement, we qualitatively assessed the motivation of healthcare workers to document their medical activities.

## Methods

### Setting

This study took place at University Hospital Zurich (USZ), Zurich, Switzerland, a 950-bed tertiary-care teaching hospital providing all medical specialties except paediatrics and orthopaedics, featuring six intensive care units (ICU) of which one is a burn unit, various organ transplant units, and a hematopoietic stem cell transplantation unit. The hospital has an IP program with an interprofessional team of 20 members to sustain prospective surveillance for surgical site infection (SSI), catheter-associated urinary tract infection (CAUTI), ventilator-associated infection (VAP), and non-ventilator-associated pneumonia, and catheter-related bloodstream infection (CRBSI), teaching, promotion, outbreak investigation and control, and research. As a unique feature, the hospital leadership committed to an institutional goal to decrease HAI prevalence from initially 8.7% in 2013 to under 5.0% in 2018.

### Study design

The quantitative approach in this mixed-methods study was a transversal assessment of assessing EMR documentation status regarding prevention measure execution in a defined inpatient population from annual point prevalence studies (PPS). The qualitative approach used semi-structured interviews and a two-phase inductive-deductive qualitative analysis.

### Quantitative investigation

#### Prevalence studies

The study included all patients enlisted in the four annual PPS from 2013 to 2016 who encountered one of the four major procedure-related HAI, i.e., SSI, CAUTI, VAP, and CRBSI according to the European Centre for Disease Prevention and Control (ECDC) HAI definitions [5]. The following variables were automatically extracted from the electronic patient record system: patient identity and case number, year of birth, sex, date of admission, transfer from another hospital (yes/no), department and unit attribution. Therapeutic antibiotic treatment (yes/no), immunosuppression (yes/no), presence of an invasive procedure, i.e., peripheral venous catheter, central venous catheter, urine catheter, tracheal ventilation tube, surgery within the last 30 days or within the last year in case of implant placement (yes/no), were extracted manually from the electronic patient records. HAI were noted with type of infection, date of onset, if acquired in USZ. These manual data extraction and determination of the HAI diagnosis were executed by trained and validated members of the IP team. The EMR covered the entire medical documentation.

#### Infection prevention standards

The in-house IP standards were developed and continuously adapted according to an established formal process. They were established based on the most recent international IP guidelines by the IP team, then submitted by the IP committee after discussion and democratic vote to the medical and nursing directorates who declare them as binding for the entire institution. The standards were published in the hospital’s intranet and accessible to every collaborator. In the remainder of the text, we will use ‘IP standards’ to refer to the IP prevention measures corresponding to the above description.

#### Documentation status definitions

For each of the included HAI, the documentation and execution status of all corresponding institutional IP standards was extracted. We opted for the 2016 version of the IP standards for their conciseness. This version listed for SSI, CAUTI, VAP, CRBSI, and overall, 15, five, 13, 23, and 56 IP standards, respectively. The documentation status in the EMR was defined as ‘*Conclusive documentation’*, i.e., if conclusive data were found in the EMR that allowed to judge whether the corresponding IP standard was executed or NOT executed with sub-status of ‘*Proof for adherence* ‘, i.e., conclusive data in the EMR that the IP standard was executed, or ‘*Proof for NON-adherence*', i.e., written proof in the EMR that the IP standard was NOT executed, and ‘*NO documentation’*, i.e., no data in the EMR that would have allowed to judge if the IP standard was executed or NOT executed.

#### Data extraction and verification

One researcher (AH) extracted the data into a dedicated Microsoft Access 2016 (Redmond, WA) database between October and December 2016 from the USZ electronic patient record system. In case of more than one HAI, only the first was included. A senior infectious diseases and IP consultant overviewed the process (SPK). A second senior consultant of the same medical specialisation (HS) verified 30% of a randomly selected sample of cases. In case of disagreement with classification, the case was discussed and resolved. These discussions and decisions served to consecutively adapt the 70% remaining cases.

### Qualitative research

To learn more about the motivation of healthcare providers to document patient care and—more specifically—IP standards, we undertook a qualitative research investigation based on semi-structured interviews and an interview guide targeting SSI, CAUTI, VAP, and CRBSI (Appendix Table 1). A purposive sample of physicians and nurses from general wards, operating theatres, ICUs, and intermediate care units were interviewed to gather a broad range of experiences. The sample size was determined by saturation for new findings [6]. All interviews were conducted by one researcher (AH) in Swiss-German or German, recorded and transcribed as paraphrasing for the majority of content and verbatim for relevant quotes. Two researchers (AH, HS) performed a two-step content analysis of the transcripts. The first step produced inductive codes following grounded theory [7]. Starting with a first interview transcript, the inductive coding progressed with the following transcripts establishing a code-book—with iterative re-coding of former interviews where necessary. The codes were then grouped into themes. In a second step the themes were deductively ordered and described according to the three main domains of the Theory of Planned Behaviour (TPB), the *Attitude*, i.e., the degree to which a person sees the anticipated outcome of the behaviour as favourable or unfavourable, *Subjective Norm*, i.e., a person’s view of whether important others approve or disapprove of the behaviour, and *Perceived Behavioural Control*, i.e., a person’s subjective perception of ease or difficulty to perform the behaviour, including factual facilitators and barriers [8, 9]. These three domains act, according to the TPB, as determinants on the *Intention* to execute the behaviour, which in turn is the unique determinant to *Act*, i.e., execute the behaviour.

## Results

### Quantitative investigation results

Overall, 2972 patients were included, i.e., 699, 717, 784, and 772 in the study years, respectively. Overall, 249 patients had 272 HAI corresponding to a prevalence of patients with any HAI of 8.4% (Appendix Table 2). Of these, 116 (47%) patients matched our inclusion criteria and encountered 78 SSI (67%), 21 CAUTI (18%), 10 VAP (9%), and 7 CRBSI (6%).

**Fig. 1:**
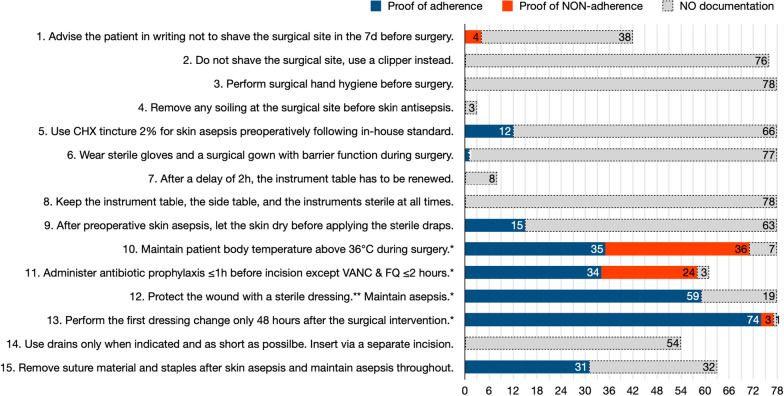
Documentation of surgical site infection (SSI) prevention standard adherence. Ad 1., in 36 cases, the decision to operate was taken less than 7 days before the procedure; ad 2., in two cases, no hair removal was necessary; ad 4., only three cases had noticeable soiling on the surgical site; ad 5., in 12 cases, adherence to standard was documented, in 66 other cases, nothing was documented; ad 7., only eight cases had a waiting time of ≥ 2 h; ad 11., in 17 cases, antibiotic prophylaxis was not indicated; ad 14., in 24 cases no drains were inserted; ad 15., in 15 cases, an absorbable suture was used. *Infection prevention standards with ≥ 75% conclusive documentation. **Accepted sterile dressings: transparent film, gauze, or fleece. VANC, vancomycine; FQ, fluorochinolones

**Fig. 2:**
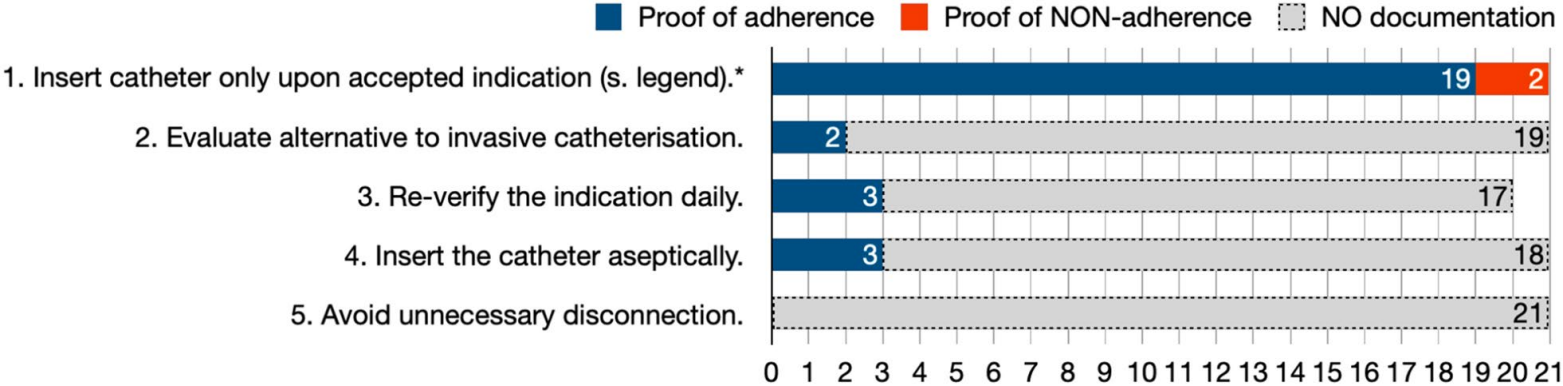
Documentation of catheter-associated urinary tract infection (CAUTI) prevention standard adherence. Ad 1., list of institutionally accepted indications: urinary retention, urine monitoring/balancing, surgery, prolonged immobilization, decubitus ulcers in case of incontinence, comfort in case of palliation; Ad 3., one patient had a duration of only one day of catheterization. *Infection prevention standards with ≥ 75% conclusive documentation

**Fig. 3:**
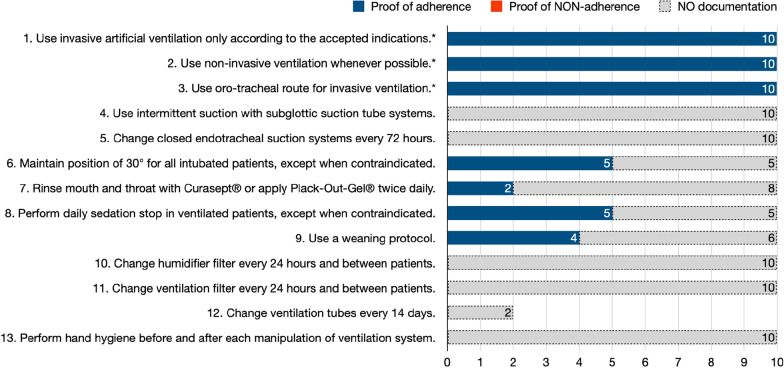
Documentation of ventilator-associated pneumonia (VAP) prevention standard adherence. Ad 1., institutional list of accepted indications: respiratory failure with profound unconsciousness with failure of protective reflexes, obstruction or swelling of the upper airway, clinical fatigue, Inadequate work of breathing with deterioration of gas exchange, respiratory insufficiency with deterioration of gas exchange; ad 6., institutional list of accepted contraindications to bed elevation rule: circulatory instability, instable pelvic or spinal injury, craniocerebral and other neuro-intensive medical conditions, modified according to cerebral perfusion pressure; ad 12., only two cases were ventilated for more than 14 days. *Infection prevention standards with ≥ 75% conclusive documentation

**Fig. 4:**
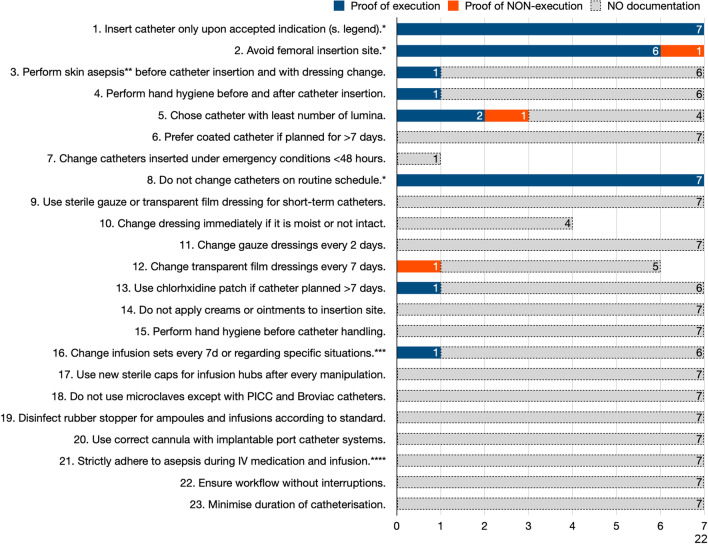
Documentation of catheter-related bloodstream Infection (CRBSI) prevention standard adherence. Ad 1., list of institutionally accepted indications: prolonged administration of circulatory drugs, administration of high osmolar substances, administration of infusions and drugs irritating the veins, measurement of venous O_2_ and pressure, semi-recumbent position in neurosurgery (air embolism prophylaxis), foreseeable intravenous therapy of > 2 weeks if peripherally inserted central catheter (PICC) contraindicated, very difficult vein conditions and repeated punctures; ad 7., only one catheter was inserted in emergency situation; ad 10., three patients did not fulfil the criteria of moist or non-intact dressing; ad 12., in one case, no film dressing was used. *Infection prevention standards with ≥ 75% conclusive documentation. **Skin asepsis requires chlorhexidine/alcohol 2%. ***Specific situations were: 6 h after transfusion; every 8 h with lipid solution; every 24 h with parenteral nutrition. ****This includes not speaking or wearing a mask. PICC, peripherally inserted central catheters

We found *conclusive documentation* to IP standards for SSI, CAUTI, VAP, CRBSI, and overall, in 328/931 (35%), 29/104 (28%), 46/122 (38%), 29/151 (19%), and 432/1308 (33%) cases, respectively; *proof of adherence* in 261/931 (28%), 27/104 (26%), 46/122 (38%), 26/151 (17%), and 360/1308 (28%) cases, respectively; *proof of NON-adherence* in 67/931 (7%), 2/104 (2%), 0/122 (0%), 3/151 (2%), and 72/1308 (6%) cases, respectively (Figs. 1, 2, 3 and 4). The number of standard items with ≥ 75% documentation for SSI, CAUTI, VAP, CRBSI, and overall, was four (27%), one (20%), three (23%), three (13%), and 11 (20%), respectively (Figs. 1, 2, 3 and 4).

### Qualitative research results

Saturation was reached with 19 interviewees; eight nurses (eight female), 11 physicians (five female); nine from floor ward (seven female), three from ICU (three female), one from intermediate care unit (one female), and six from operating room (two female), all between ~ 25 and ~ 35 years of age. Of the 67 inductive codes, 40 were allocated to the TPB dimension A*ttitude* with 148 coded interviewee statements (snippets), four to *Subjective Norm* with 78 snippets, and 23 to *Perceived Behavioural Control* with 491 snippets.

#### Attitude

According to the TPB, an individual's *Attitude* towards an action is the product of various positive or negative behavioural beliefs about what results from a given action, while the *Attitude* represents an antecedent of the *Intention* to act, i.e., to document in our case [9]. We found the following *Attitudes* towards documentation (with themes highlighted in bold).

Documentation is meant to guarantee the **continuity of care** in the case of patient handovers between physicians or care teams.

*“Each therapeutic decision should be noted in a way so that if the treating physician falls ill the next day, one can understand why something has been changed.”* (Male resident, floor ward)

Documentation can also serve as a **reminder** for themselves or for their colleagues.

*"To remember why the patient has one [invasive device]."* (Male resident, floor ward)

*"*[To] *remind yourself, oh what did I* [do] *again yesterday on this patient and then you can read up. For your own history, but also for night shifts."* (Female resident, floor ward)

Some healthcare providers consider documentation as a **safeguard against legal consequences**.

"In this profession you have one foot in prison, (laughs) by now." (Female resident, floor ward)

However, on the contrary, the **fear of negative (legal) consequences** was also a reason to abstain from documentation, in order to avoid evidence of one's own misconduct.

*"In the case of a secondary infection, you don't want the report to say that you made the operating field unsterile, but then continued to work anyway. Then you dig your own grave."* (Male resident, floor ward)

Other reasons against documentation were **repetitions**, which seem to weaken the motivation to document a medical procedure in writing.

*“If the indication* [for a urinary catheterization] *leads to a prescription for several days, the re-evaluation will not be documented every day. For example, it is clear that the prostate volume will not get so small in one day that it would allow us to take the catheter out.”* (Female resident, floor ward)

Finally, a **perceived lack of relevance** was the most frequent answer to the question why a certain action should or should not be documented.

*"I think it's unnecessary. Not only because of the time, but simply because it (pause 3 seconds) makes no sense."* (Female resident, floor ward)

In general, healthcare providers said they do not document the **course of an action** but limit themselves to documenting the result.

*"It's really just like when the process is done, the result where you write it down. You don't write down the workflow."* (Female nurse, floor ward)

#### Subjective Norm

Human behaviour is considerably influenced by the perceived judgement of one’s actions by subjectively important others. As a physician resident points out, a “laissez-faire attitude of superiors” can impair the necessary discipline to document.

**Professional pride** seems to positively influence the quality of documentation.

*“The documentation by nurses is a picture of our care*—*concise, using technical terms, precise.”* (Female nurse, floor ward)

Interestingly, even the **design of digital interfaces** can transport the message that documentation is necessary and expected.

*“If it is visible* [on the screen] *and one can only select it by a click it will be used because it suggests that it could have a legal relevance.”* (Female resident, operating room)

On the other hand, young residents in particular wonder what the **senior physician will think of them** if they go into too much detail in their account of procedures.

*"The one who co-signs the report would probably delete it if I had documented it."* (Male resident, floor ward)

The decision not to document is apparently governed by the assumption that a medical procedure is carried out according to an established ‘standard’. Only **deviations from the norm** would be noted.

*“Hand hygiene and all these things that are, like, self-evident are not documented. Except if something goes wrong, the patient touches the wound, then yes.”* (Female nurse, floor ward)

*"It would make sense to document when you have deviated from the internal standard and give a reason why."* (Female resident, operating room)

Especially **actions** that appear to be **self-evident** in the eyes of the community are not documented.

*“I see this* [hand hygiene] *as something personal, and so, very self-evident.”* (Male resident, operating room)

#### Perceived behavioural control

The third antecedent of the *Intention* to *Act* in the TPB reflects the control one has over one’s capability to execute planned actions. This includes both, perceived but also real barriers and facilitators to execution.

The time needed for documentation depends on the **design of the documentation process**. Many interviewees expressed their frustration with bad information technology interfaces, or their ideas and desire for better systems, especially through automatization.

*"I would document much more if I had voice recognition software."* (Female resident, floor ward)

**Time restraints** count among the most frequently cited barriers to go and write a procedure down.

*"Lack of time is of course the biggest obstacle."* (Female resident, ICU)

The **lack of consistent digitalization** was often criticized in the context of documentation.

*“The results of an electronic arterial blood pressure measurement have to be noted on a paper slip and from there, they have again to be typed into the* [EMR] *system by hand.”* (Female nurse, floor ward)

*“Many different documentation systems lead to you forgetting it and it is very cumbersome.”* (Female nurse, floor ward)

A **noisy work environment** can jeopardize work in general but also interfere with the quality of documentation.

*“Too many people in one room, I cannot concentrate!”* (Female nurse, floor ward)

## Discussion

This study investigated the documentation of adherence to IP standards in individual EMRs. It became evident that there was no mandatory institutional practice to deliberately document IP standards. In consequence, the status of IP standard adherence had to be evaluated based on data that were registered in EMRs for other reasons. Even so, we found the degree of documentation to be poor. The qualitative research corroborated and explained these findings: IP standards do only rarely fall in the category of topics for which healthcare providers see value in documentation, there is no cultural impetus to document them, and EMR interface design does not facilitate documentation in general.

Our findings are not entirely surprising. Infection prevention measures are not considered as primary medical tasks. This might lead to the notion that their documentation is not necessary. In the last two decades, however, some of these prevention measures have become subject to systematic monitoring, such as the adherence to the WHO “My five moments for hand hygiene” [10, 11], the proportion of patients receiving antibiotic prophylaxis within one hour before the incision [12], and the proportion of patients with a urinary catheter with an accepted indication [13]. These measurements, however, are usually captured and reported at the population level and are not part of the individual EMR. Hand hygiene adherence for example is mostly collected and reflected as a quality attribute of the institution [10] and not documented from the individual perspective of the patient.

The degree of available data on IP standard adherence varied largely across individual standards and type of infection. IP standards with ≥ 75% documentation were found regarding the indication for invasive central vascular lines, urinary catheters, and ventilation tubes in the context of CAUTI, VAP, and CRBSI. Additionally, for central lines, the standard ‘Don’t change lines on a regular basis’ could be inferred from the irregular change intervals in the nursing charts. VAP was the HAI with the highest proportion of conclusive documentation. There, adherence to the list of approved indications, use of non-invasive ventilation, and use of oro-tracheal route were among the standards with ≥ 75% documentation. With SSI, body temperature, antibiotic prophylaxis, and dressing handling were among the standards with ≥ 75% documentation. While we found no unequivocal principle behind determinants for documentation of IP standard adherence, there are some partially overlapping categories to be addressed. First, there is the documentation of indications for invasive medical procedures such as inserting urinary catheters, central vascular lines, intubation, etc. These procedures are typically noted in the EMR due to their invasive nature and potential legal implications and therefore, generally met a high proportion of documentation. Second, a category also meeting a fair proportion of documentation concerned routines that are traditionally noted in the nursing chart or correspond to medical prescriptions, e.g., daily sedation stops, weaning protocol, oral hygiene in ventilated patients, dates of changing central lines, preoperative administration of antibiotic prophylaxis, body temperature during surgery. Interestingly, the most frequent documentation of non-adherence to IP standards appeared with the latter two examples of objective technical measurements. Third, there are practices that are usually decided on an institutional level, e.g., changing humidifier filters every 24 h, use of coated catheters in long-term central lines, not to use a razor for preoperative hair removal, general use of subglottic suction ventilation tubes, use of sterile gaze or transparent film dressing for short-term catheters, performance of hand hygiene before surgery, ban to use ointments on catheter insertion sites, and use of new sterile caps after infusion hub manipulation. We found this category to be barely documented in the individual EMR, which was explained by interviewees as “too obvious” to note because these things are “supposed to happen”. We know, however, from a large body of literature (for example on adherence to hand hygiene) that institutional rules sometimes meet a surprisingly low adherence and do not ‘just happen’. An exception to the described pattern is noteworthy, regarding the rule of 30° headrest elevation. At the time of the study, this was part of an intervention to reduce VAP and was monitored through established documentation [14]. Fourth, it was sometimes impossible to infer IP standard adherence from EMR data due to lacking detail in the description of a specific process, e.g., the use of sterile gloves and gowns during surgery, the insertion of drains through a separate incision, and the performance of hand hygiene before and after insertion of a catheter. Fifth, there are ways of working that are habitual due to their frequent occurrence in various contexts, e.g., ensuring uninterrupted workflows, correct hand hygiene during patient care, minimal duration of catheterization and would drains, keeping the operating table sterile, avoiding unnecessary disconnection of urinary catheters, considering alternatives for catheterization. EMR documentation in this category was also rare.

We performed qualitative research to investigate the views and motivations of healthcare providers regarding documentation of IP standards and medical information in general. The results support and explain the quantitative findings. They reflect a current reality of suboptimal EMR documentation interfaces and processes in the context of a challenging work reality that limits the will to document to subjects of recognizable practical benefit. This insight holds promise. Modifiable checklists and registers, the ability to easily design and run reports, and user-friendly provider interfaces have been identified as key elements to promote effective use of the EMR [15]. Page and Schadler showed that the time to complete a document decreased and staff satisfaction increased significantly among nursing staff after redesigning the EMR with a focus on usability [16]. Such structure design modifications hold the greatest promise for success according to the hierarchy of intervention effectiveness [17]. Zahabi et al. recommended guidelines for EMR interface design to increase documentation quality [18]. Additionally, a possible but radical idea to change the perceived benefit for documentation (i.e., positive *Attitude*) could be to inform patients at their admission about the institution's IP standards and provide them with an account of adherence at their discharge. Obviously, this would have to be accompanied by a profound error and safety culture change towards openness (i.e., *Subjective norm*) and facilitating documentation infrastructure and process (i.e., *Perceived and actual behavioural control*) [4].

While there are myriads of reports on monitoring of IP behaviour on hand hygiene, none of these discuss the possibility to specifically document hand hygiene execution in the individual EMR. This is surprising when considering that newer automated hand hygiene monitoring systems would lend themselves to measure patient-centric data and transfer the achieved overall quality automatically into the EMR [19]. We believe that automated registration of IP adherence-relevant data—including through sensor technology—holds a specially promising potential to capture patient-level data in general because of its independence of human desirability bias. Timely feedback of such data might not only be interesting for the patient, but would likely give healthcare providers a novel, empathic view of their IP behaviour. But conscious documentation of IP standard adherence—or non-adherence—in the EMR would still be beneficial to raise awareness of these procedures that are often forgotten or taken for granted. To this end, the EMR data entry should be structured and user-centred to avoid unnecessary additional workload. This requirement for better design extends to the IP standards that should be created as one cycle including policy, execution, documentation, and feedback.

This study has limitations. It took place in a single institution in a confined period and patient population. In particular, the inclusion of patients with HAI might have introduced a bias regarding documentation quality. However, we estimate that this bias would have been minimal since the later occurrence of a HAI could not have been anticipated. While this limits generalizability, we think that the findings nevertheless aptly raise the fundamental question of the value and challenges of IP standard documentation on the patient level. The study design did not, however, explore the question whether a more comprehensive documentation would have a positive influence on adherence to IP measures. It also remains unclear if a full disclosure of non-adherence would ever be possible in the current legal and cultural landscape. Neither did we question the respective effect of individual IP standards on infectious risks. These questions would warrant further investigation.

## Conclusions

This in-depth investigation found that deliberate documentation of adherence to IP standards in EMRs was not an established process and conclusive data for adherence was infrequent. Conclusive indirect proof was mainly associated with invasive medical procedures and traditionally established routine chart data. The qualitative research confirmed that patient-level documentation of IP standards does not fall in the category of topics for which healthcare providers see value in documentation, there is no cultural impetus to document them, and EMR interfaces do not facilitate documentation in general. Based on these results we hypothesise that improved EMR interface design and automatic capturing and integration of routine IP behaviour could bridge this gap. We also pose that disposing of patient-level IP data would most likely result in increased awareness of IP and help to advance patient safety—and should become an institutional standard.

## Data Availability

Data is available from the corresponding author upon request.

## Appendix

See Appendix Tables 1 and 2.

**Appendix Table 1:** Semi-structured interview guide

HAI	Healthcare worker	Questions
SSI	Resident, floor ward (Surgeon)	What was the last operation you performed? Tell me what you have to consider before the operation and what you documented afterwards?
		What was the basis for the decision to document?
		Do you prescribe the antibiotic prophylaxis or the anaesthesiologist itself?
		What and how is this documented?
		Did you perform the operation yourself?
		Are minor details also documented? E.g. hand disinfection or if you reach somewhere with the swab and it would no longer be aseptic?
		Can you tell me your reasoning for documenting something?
		And why did you document it that way (why not more or less?)?
		Does it happen that something is not (or not fully) documented? Why do you think it is not documented?
		What are generally barriers and facilitators to documentation?
	Resident, operating room (Anaesthesiologist)	What was it like the last time you attended an operation? Tell me what you did and documented afterwards?
		How do you deal with the prescribed antibiotic prophylaxis?
		What do you document regarding your involvement with it?
		Are minor details also documented? E.g. hand disinfection or if you get somewhere with your clothes on and it would no longer be aseptic?
		Can you tell me your reasoning for documenting something?
		And why did you document it that way (why not more or less?)?
		Does it happen that something is not (or not fully) documented? Why do you think it is not documented?
		What are generally barriers and facilitators to documentation?
	Nurse, floor ward	What was it like the last time you took care of a surgical wound? Tell me what you did and documented afterwards?
		Are minor details also documented? E.g. hand disinfection or if you reach somewhere with the swab and it would no longer be aseptic?
		Can you tell me your reasoning for documenting something?
		And why did you document it that way (why not more or less?)?
		Does it happen that something is not (or not fully) documented? Why do you think it is not documented?
		What are generally barriers and facilitators to documentation?
CAUTI	Resident, floor ward	Do you deal with urinary catheters?
		When was the last time you prescribed a urinary catheter? Tell me what you did and documented afterwards?
		What was the basis for the decision to document the initial indication?
		Is the decision process regarding the alternatives documented?
		What was the basis for the decision to document the follow-up indication?
		What is documented regarding the insertion?
		Are minor details also documented? E.g. hand disinfection or if the catheter is placed somewhere and it is no longer aseptic?
		What is the basis for the decision to document a deconnection?
		Can you tell me your reasoning for documenting something?
		And why did you document it that way (why not more or less?)?
		Does it happen that something is not (or not fully) documented? Why do you think it is not documented?
		What are generally barriers and facilitators to documentation?
	Nurse, floor ward	Do you deal with urinary catheters?
		What was it like the last time you inserted a urinary catheter? Tell me what you did and documented afterwards?
		What is documented regarding the insertion?
		Are minor details also documented? E.g. hand disinfection or if the catheter is placed somewhere and it is no longer aseptic?
		What is the basis for the decision to document a deconnection?
		Can you tell me your reasoning for documenting something?
		And why did you document it that way (why not more or less?)?
		Does it happen that something is not (or not fully) documented? Why do you think it is not documented?
		What are generally barriers and facilitators to documentation?
VAP	Resident, ICU	What was it like the last time you did an intubation? Tell me what you did and documented afterwards?
		What was the basis for the decision to document the initial indication?
		Is the decision-making process regarding the alternatives documented?
		What is then documented regarding the intubation?
		Are minor details also documented? E.g. hand disinfection or if you get somewhere with the tube and it would no longer be aseptic?
		How is the elevation of the upper body documented?
		How is the sedation stop documented?
		Can you tell me your reasoning for documenting something?
		And why did you document it that way (why not more or less?)?
		Does it happen that something is not (or not fully) documented? Why do you think it is not documented?
CRBSI	Resident, floor ward	Are you dealing with CVCs?
		How was it the last time you prescribed a CVC? Tell me what you did and documented afterwards?
		What was the basis for the decision to document the initial indication?
		How is the prescription of the insertion site documented?
		How is the prescription of the catheter documented?
		What was the basis for the decision to document the follow-up indication?
		Can you tell me your reasoning for documenting something?
		And why did you document it that way (why not more or less?)?
		Does it happen that something is not (or not fully) documented? Why do you think it is not documented?
		What are generally barriers and facilitators to documentation?
	Resident, operating room (Anaesthesiologist)	Are you dealing with CVCs?
		What was it like the last time you inserted a CVC? Tell me what you did and documented afterwards?
		What do you document regarding your consideration of the initial indication?
		What is documented regarding the insertion?
		Are minor details also documented? E.g., hand disinfection or if the catheter is placed somewhere and it is no longer aseptic?
		Can you tell me about your reasoning for documenting something?
		And why did you document it that way (why not more or less?)?
		Does it happen that something is not (or not fully) documented? Why do you think it is not documented?
		What are generally barriers and facilitators to documentation?
	Nurse, floor ward	Are you dealing with CVCs?
		What was it like the last time you took care of a CVC in a patient? Tell me what you did and documented afterwards?
		What is documented regarding the care?
		Are minor details also documented? E.g. hand disinfection or if you reach somewhere with the swab and it would no longer be aseptic?
		Can you tell me your reasoning for documenting something?
		And why did you document it that way (why not more or less?)?
		Does it happen that something is not (or not fully) documented? Why do you think it is not documented?
		What are generally barriers and facilitators to documentation?

**Appendix Table 2:** Patient characteristics according to infection status in four point-prevalence studies 2013–2016

	Overall	With any type of HAI	With one of the four included HAI types (study population)
N	2972	249	116
Median age in years (range)	60 (2–98)	64 (19–94)	64 (19–93)
Female sex (%)	1430 (48.1)	99 (39.8)	49 (42.2)
Median length of hospital stay in days (range)	10 (1–366)	31 (2–221)	30 (2–209)
*Patient groups*			
Surgery (%)	1072 (36)	142 (56.2)	86 (74.1)
Internal Medicine (%)	883 (30)	53 (21.3)	17 (14.7)
Onco-haematology (%)	249 (8)	39 (15.7)	2 (1.7)
Gynaecology & Obstetrics (%)	456 (15)	13 (5.2)	9 (7.8)
Ophthalmology, Dermatology, ENT, Radio & Nuclear (%)	312 (10)	4 (1.6)	2 (1.7)
*Treatment/installations*			
Antibiotic treatment (%)	966 (32.5)	233 (93.6)	110 (94.8)
Peripheral venous catheter (%)	1325 (44.6)	119 (47.8)	57 (49.1)
Central venous catheter (%)	552 (18.6)	126 (50.6)	58 (50)
Urinary catheter (%)	590 (19.9)	88 (35.3)	42 (36.2)
Intubation (%)	136 (4.6)	34 (13.7)	17 (14.7)
Immunosuppressed (%)	330 (11.1)	62 (24.9)	19 (16.4)
Surgery within the last 30 days (%)	1321 (44.4)	151 (60.6)	91 (78.4)
Surgery with implant within the last year (%)	440 (14.8)	73 (29.3)	47 (40.5)

## Abbreviations

CAUTI: Catheter-associated urinary tract infection
CRBSI: Catheter-related bloodstream infection
EMR: Electronic medical record
HAI: Healthcare-associated infections
ICU: Intensive care unit
IP: Infection prevention
PPS: Point prevalence studies
SSI: Surgical site infection
VAP: Ventilator-associated pneumonia
